# Clinical analysis of 37 Chinese patients with ocular amyloidosis: a single center study

**DOI:** 10.1186/s12886-024-03548-w

**Published:** 2024-07-18

**Authors:** Jing Li, Rui Liu, Tingting Ren, Nan Wang, Qihan Guo, Liangyuan Xu, Jianmin Ma

**Affiliations:** Beijing Institute of Ophthalmology, Beijing Tongren Eye Center, Beijing Tongren Hospital, Capital Medical University, Beijing, 100730 China

**Keywords:** Amyloidosis, Orbital disease, Conjunctiva, Clinical presentation, Treatment

## Abstract

**Objective:**

To examine the clinical characteristics, diagnosis and treatment, and prognosis of ocular amyloidosis in a Chinese population.

**Methods:**

A retrospective case series study was conducted. The clinical data of 37 patients with ocular amyloidosis were collected and the clinical characteristics, diagnosis and treatment, and prognosis were summarized and analyzed.

**Results:**

The 37 patients included 12 males and 25 females ranging in age from 22 to 75 years, with median age of 49 years. The clinical signs and symptoms included a conjunctival mass in 37 patients (100%), periorbital discomfort or pain in 29 patients (61.9%), ptosis in 18 patients (23.8%), exophthalmos or eyeball displacement in 3 patients (14.3%), restricted eye movement in 2 patients (9.52%), vision loss in 1 patient (4.76%), and diplopia in 1 patient (4.76%). A total of 29 patients had only conjunctival involvement and 8 patients had concomitant orbital and conjunctival involvement. The main treatment for patients with conjunctival involvement was surgical resection. Thirty-one patients had stable disease, 4 patients progressed or relapsed, and 2 patients were lost to follow-up.

**Conclusion:**

Ocular amyloidosis most commonly presents as an eyelid or conjunctival mass or diffuse thickening and can also present as an orbital mass. Diagnosis is mainly dependent on histopathological examination. Surgery is the main treatment and is done to confirm the diagnosis to guide further treatment, preserve function, and prevent complications that threaten visual acuity. Close postoperative follow-up is necessary.

## Keypoints

• Ocular amyloidosis most commonly presents as an eyelid or conjunctival mass or diffuse thickening and can also present as an orbital mass.

• Diagnosis is mainly dependent on histopathological examination. Surgery is the main treatment and is done to confirm the diagnosis to guide further treatment, preserve function, and prevent complications that threaten visual acuity.

## Introduction

Amyloidosis is a disease caused by the deposition of insoluble amyloid protein in tissues and organs [1]. Amyloidosis can be classified as systemic or localized based on the disease site and as primary or secondary based on etiology [2]. Primary systemic amyloidosis is the most common form. Almost all ocular structures can be affected, including the eyelids, conjunctiva, eye socket, cornea, and vitreous humor. However, misdiagnosis and missed diagnosis by ophthalmologists is common because of the low incidence of amyloidosis and the lack of a specific clinical presentation. In order to further understand and explore the diagnostic basis and improve the diagnosis rate of this disease, the clinical data of 37 patients who were pathologically confirmed to have ocular amyloidosis and treated in our hospital between March 2004 and March 2022 were collected; the clinical characteristics, diagnosis and treatment, and prognosis were summarized and analyzed.

## Data and methods

Thirty-seven patients were diagnosed with ocular amyloidosis at the Ocular Oncology Department of Beijing Tongren Hospital between March 2004 and March 2022. All patients underwent surgery, were diagnosed with amyloidosis by histopathology, and had complete medical records. The medical record data, disease course, clinical presentation, involved sites, radiologic presentation, laboratory tests, pathological characteristics, type of surgery, and prognoses were reviewed. All patients had undergone slit-lamp examination for anterior segment and fundoscopic examination to assess posterior segment conditions.

## Results

### General status

The 37 patients included 12 males and 25 females ranging in age from 22 to 75 years, with a median age of 49 years. It was more common in women and the median age of onset was 51 years. Thirty-three patients had monocular disease and 4 patients had bilateral disease. Four patients with both eyes were affected only with localized lesions and no systemic lesions. The mean duration from onset of clinical signs and symptoms to diagnosis of amyloidosis was 95 months (median: 44, range: 1–43 years). Thirty-five patients denied a history of autoimmune disease, inflammatory bowel disease, chronic eye infection or inflammation, or a family history of amyloidosis. One patient had been diagnosed with systemic amyloidosis 3 years prior and with Sjogren’s syndrome 2 years prior to being seen at our hospital. This patient had undergone tracheal lesion resection surgery at another hospital and the postoperative pathology test showed amyloidosis. One patient had been diagnosed with systemic amyloidosis 17 years prior to being seen at our hospital. This patient had undergone right intraorbital mass resection surgery due to bilateral eyelid swelling and exophthalmos at another hospital; the postoperative pathology test showed amyloidosis. Patients with systemic amyloidosis typically undergo chemotherapy with Bortezomib + Dexamethasone.

#### Clinical presentation

The clinical signs included a conjunctival mass in 37 patients (100%), periorbital discomfort or pain in 29 patients (78.4%), ptosis in 18 patients (48.6%), eyelid swelling in 13 patients (35.1%), subconjunctival bleeding in 4 patients (10.8%), exophthalmos or eyeball displacement in 3 patients (8.1%), restricted eye movement in 2 patients (5.4%), vision loss in 1 patient (2,7%), and diplopia in 1 patient (2.7%).

**Fig. 1:**
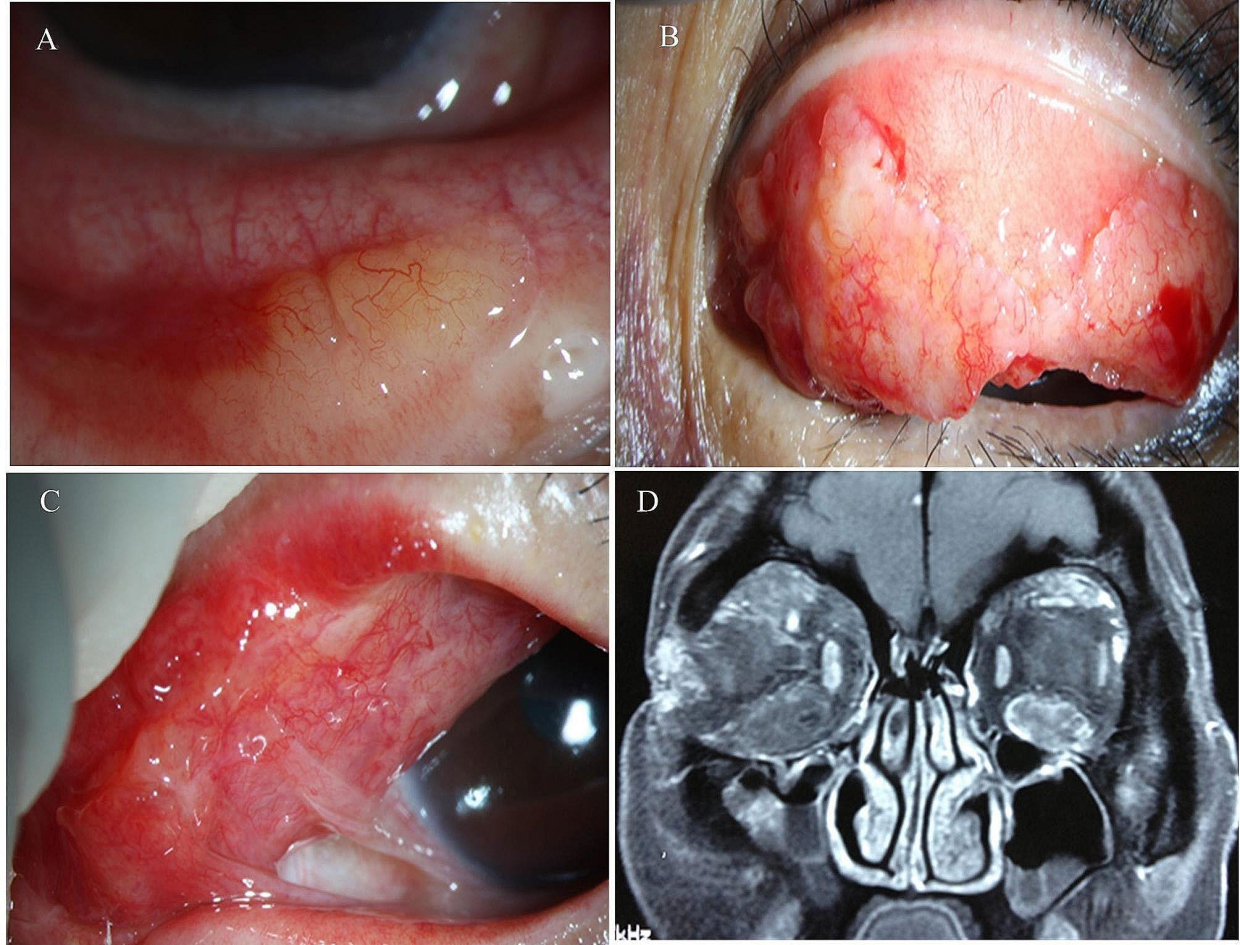
Clinical and imaging findings of ocular amyloidosis. **A**. A yellowish nodular mass in the conjunctiva of the right eye. **B**. Cauliflower-like mass in the conjunctiva of the left eye. **C**. The mass is pink involving the palpebral conjunctiva and/or fornix conjunctiva. **D**. The right superior ocular muscle group, the inferior rectus muscle and the upper oblique muscle, the left inferior rectus muscle and the superior ocular muscle group were significantly thickened

In the 29 patients with only conjunctival involvement, 26 patients had unilateral diffuse eyelid thickening and a hard nodular mass could be palpated, which was pink, of moderate hardness, granular, cauliflower- or okara-like in appearance, and involved the palpebral conjunctiva and/or fornix conjunctiva (Fig. 1A-C). Three patients presented with a bilateral nasal bulbar conjunctiva and semilunar line pink mass that was brittle and had a fish flesh-like appearance. No apparent abnormalities were observed in 29 patients upon preoperative physical examination.

No abnormalities were observed upon examination for systemic amyloidosis and no amyloid deposits were found in a second biopsy site in 5 of the 8 patients with concomitant orbital and conjunctival involvement, whereas 3 of the 8 patients had systemic light chain amyloidosis.

#### Orbital magnetic resonance imaging (MRI) examination

The 8 patients with concomitant orbital and conjunctival involvement underwent orbital MRI examination. The results showed orbital and lacrimal involvement in 3 patients; extraocular muscle involvement in 1 patient; and eyelid, lacrimal gland, and extraocular muscle involvement in 4 patients. All signals of lesions were uneven long T1 and short T2 signals and exhibited mild contrast enhancement (Fig. 1D).

#### Pathological presentation

**Fig. 2:**
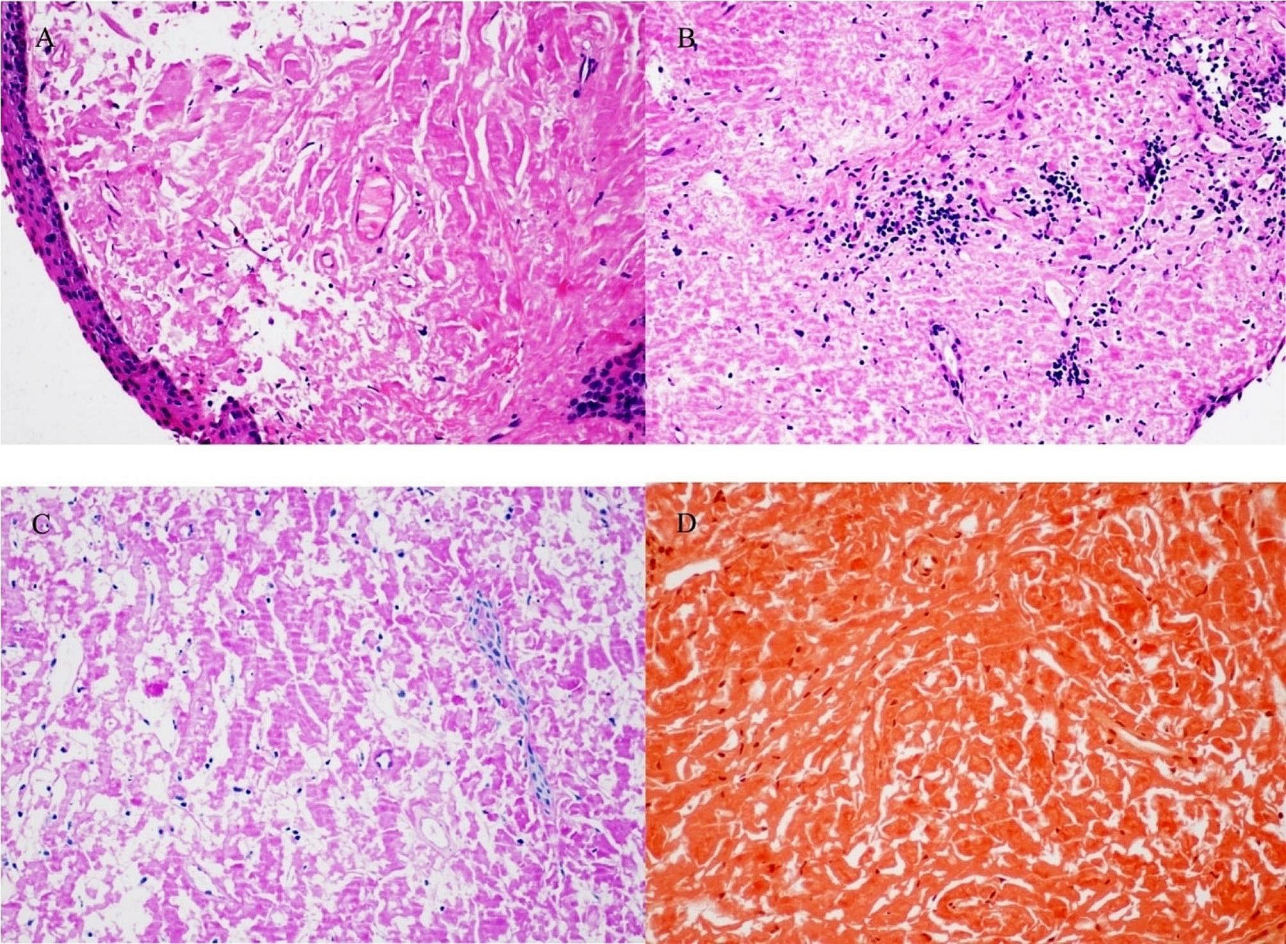
Histopathological manifestations of conjunctival amyloidosis (magnification X200). **A**. The mucous epithelium showed powdery clouded, flaky and even clumpy amyloid substances; **B**. Chronic inflammatory cell infiltration; **C**. The amyloid substance was pink after PAS staining; **D**. Congo red staining showed that the amyloid was pink

The 37 patients included in this study were all pathologically diagnosed by HE staining and microscopy. The samples showed diffuse degenerative lesions in that were replaced by pink, homogeneous, and amorphous substances. Vascular degeneration presented as debris-like changes that were occasionally accompanied by focal calcification. Immunohistochemical staining results showed moderately or strongly positive Congo red staining (Fig. 2).

#### Treatment and prognosis

Surgical approaches include excision and debulking. As the condition is benign, the surgical principle is primarily based on preserving the normal function of surrounding tissues while attempting to remove the lesion tissue as much as possible. If complete excision is not feasible, debulking surgery is performed. The 29 patients with only conjunctival involvement underwent mass resection surgery. Of these patients, 26 had no occurrence of relapse during follow-up, only one patient experienced a recurrence of conjunctival lesions 17 months postoperatively, and 2 were lost to follow-up. If the defect area of conjunctival tissue after lesion excision is too large to be directly sutured, autologous conjunctival transplantation technique is employed for ocular surface reconstruction. The 8 patients with concomitant orbital and conjunctival involvement underwent intraorbital lesion resection surgery. Of these patients, 5 had no occurrence of relapse during follow-up and 3 patients progressed. After surgery, three patients underwent adjuvant chemotherapy, commonly using the MP regimen (Methotrexate + Prednisone), which generally yields favorable responses. However, in one case with unsatisfactory chemotherapy response, adjuvant radiotherapy was administered.

## Discussion

Amyloidosis is a disease caused by the deposition of insoluble amyloid protein in tissues and organs. Amyloidosis can be classified as systemic or localized based on the disease site [3, 4]. Localized amyloidosis is rarely seen in clinical practice and mostly occurs in the head and neck. Only 4% of localized amyloidosis cases occur in the eye socket and mainly involve the eyelids and conjunctiva [5, 6]. Lacrimal and/or extraocular muscle involvement is rare [7, 8]. The most common etiology of localized amyloidosis is deposition of immunoglobulins secreted by plasma cell clones. This disease is often mucosa-associated, e.g., conjunctival involvement is common in the eye. Amyloidosis can be classified as primary or secondary based on the etiology. Primary systemic amyloidosis is the most common form and accounts for 70% of amyloidosis cases [9]. This form often involves the heart, liver, kidneys, skin, and other tissues and organs. Ocular involvement can also occur and mostly presents as vitreous turbidity and retinopathy [10, 11]. We carried out a literature review and found that the most common symptoms were conjunctival mass or tissue thickening, followed by ptosis, and subconjunctival bleeding [12]. These results were similar to the findings of our study in which 29 of 37 patients had only conjunctival involvement (78.4%) and 8 of 37 patients had concomitant orbital and conjunctival involvement (21.6%). The amyloidosis symptoms in the current study included conjunctival mass in 37 patients (100%), periorbital discomfort or pain in 29 patients (78.4%), ptosis in 18 patients (48.6%), eyelid swelling in 13 patients (35.1%), subconjunctival bleeding in 4 patients (10.8%), exophthalmos or eyeball displacement in 3 patients (8.1%), restricted eye movement in 2 patients (5.4%), vision loss in 1 patient (2,7%), and diplopia in 1 patient (2.7%).

The main radiologic examinations for this disease include MRI. Radionuclide labeled serum amyloid P component (SAP) is an amyloid-specific tracer. The MRI examination can estimate the area of amyloid deposits before and after treatment and determine treatment response. Different tissues present differently in MRI examinations, and the presentations are not specific; thus MRI can only be used as auxiliary examination at present [13–15].

Histopathological examination is the gold standard for diagnosing amyloidosis and Congo red staining is specific for this disease. Microscopy mainly shows amyloid deposition in the affected tissues [16]. The histopathological diagnostic criteria for extraocular muscle amyloidosis are destruction of striated muscle structure, partial or complete loss of striation, large amounts of non-uniform cloud-like deposition of eosinophilic material, purple PAS staining, orange-red Congo red staining, and green fluorescence staining under polarized light microscopy [17]. Under immunohistochemical staining, amyloid can be classified as amyloid light chain (AL), serum amyloid A (AA), transthyretin, or cystatin C [1]. The first 2 are the most common. The AL subtype is mostly seen in primary amyloidosis and is secondary in patients with multiple myeloma, while the AA subtype is mostly seen in patients with secondary amyloidosis. The AL subtype is mostly seen in middle-aged and elderly people, of which20% have comorbid multiple myeloma [18]. Every patient that has been diagnosed with ocular amyloidosis should undergo physical examination to rule out systemic amyloidosis. The basic tests for systemic amyloidosis include routine bloodwork, urinalysis, biochemistry, coagulation function, β2-microglobulin, Ig levels, 24-h urine protein, Bence-Jones protein, electrocardiography or echocardiography, and abdominal ultrasound. Systemic amyloidosis should be suspected if the patient has proteinuria, cardiomyopathy, hepatomegaly, or peripheral neuropathy. This disease is highly suspected if monoclonal proteins are detected in blood and urine immunofixation electrophoresis [19]. Amyloidosis can also be diagnosed by biopsy at a second site. Common biopsy sites include bone marrow, subcutaneous fat, and rectum [20]. In this study, all patients underwent a physical examination. A second site biopsy was performed in 8 patients with orbital involvement. Amyloidosis was found via second site biopsy in 1 patient who was then diagnosed with systemic amyloidosis. Two patients had a history of surgery for the treatment of amyloidosis in other sites. Evidence of systemic amyloidosis was not found in the remaining 5 patients.

The clinical presentation of this disease is similar to many orbital diseases such as idiopathic orbital inflammatory pseudotumor, thyroid eye disease, and lymphoma, thus attention should be paid to the differential diagnosis [21, 22]. In this study, the possibility of idiopathic orbital inflammatory pseudotumor was high on the preoperative diagnosis list in all 8 patients with orbital involvement, which supports the fact that diagnosing amyloidosis based on clinical presentation and radiologic examination can be difficult and that only histopathological testing of surgically resected tissues can be used for definitive diagnosis. One patient had a definitive diagnosis of amyloidosis after a surgery at another hospital 17 years prior to being seen at our hospital, but did not undergo systemic physical examination and treatment at that time, resulting in a worsening of the condition and affects to the patient’s physical and mental health. Therefore, early diagnosis by biopsy and early aggressive treatment are extremely important.

Amyloidosis is difficult to treat, particularly primary systemic lesions. There are 3 main aspects of treatment: (1) decreasing synthesis of amyloid substances; (2) inhibiting the accumulation and deposition of amyloid substances; and (3) promoting the dissolution of amyloid deposits. Until recently, the melphalan and prednisone (MP) chemotherapy regimen has been the standard treatment protocol since 1975 when Cohen et al. first used it to treat amyloidosis. However, only 30% of patients responded to the MP regimen according to one study [23, 24]. Other treatment measures include VBMCP (vincristine, carmustine, melphalan, cyclophosphamide, and prednisone), hematopoietic stem cell transplantation, and organ transplantation. Currently, there is no standard clinical regimen for primary lesions that are localized to the eye socket and local lesion resection and strabismus correction are mostly used [25]. A previous paper reported that employing external beam radiation therapy after surgical resection in patients with primary orbital amyloidosis resulted in good outcomes [26, 27]. However, larger studies are needed to determine the long-term efficacy of radiotherapy. In this study, the main treatment method for patients with conjunctival involvement was surgical resection. The follow-up period was 1–12 years during which 31 patients had stable disease, 4 patients progressed or relapsed, and 2 patients were lost to follow-up.

The limitation of this study lies in the small number of systemic amyloidosis cases. A more comprehensive summary requires collecting more relevant cases over a longer period. In summary, ocular amyloidosis mostly presents as an eyelid or conjunctival mass or diffuse thickening and can also present as orbital mass. Diagnosis is mainly dependent on histopathological examinations. Surgery is the mainstay treatment and is done to confirm the diagnosis to guide further treatment, preserve function, and prevent complications that threaten visual acuity. Close post-operative follow-up is necessary.

## Data Availability

We confirm that the data supporting the findings of this study are available within the article.

## Abbreviations

MRI: magnetic resonance imaging
SAP: serum amyloid P component
AL: amyloid light chain
AA: serum amyloid A
MP: melphalan and prednisone
VBMCP: vincristine, carmustine, melphalan, cyclophosphamide, and prednisone
